# A Case of Complete Heart Block and Acute Appendicitis in a Young Patient With COVID-19

**DOI:** 10.7759/cureus.22926

**Published:** 2022-03-07

**Authors:** Sherin Sallam, Claire Sullivan

**Affiliations:** 1 Department of Internal Medicine, University Hospitals Cleveland Medical Center, Cleveland, USA; 2 Department of Cardiology, University Hospitals Cleveland Medical Center, Cleveland, USA

**Keywords:** mis-c, multisystem inflammatory syndrome in children, appendicitis, third-degree av block, complete heart block, sars-cov-2, covid-19

## Abstract

Recently, coronavirus disease 2019 (COVID-19, caused by SARS-CoV-2) has posed a challenge for clinicians, particularly extrapulmonary manifestations. These manifestations are often rare and difficult to recognize as research is still underway for the myriad presentations of the disease. A 19-year-old man presented with viral upper respiratory infection (URI) symptoms and received a positive result for SARS-CoV-2 real-time reverse transcription polymerase chain testing. A few days later, he developed abdominal pain and presented to the Emergency Department (ED). He was found to have bradycardia, right lower quadrant pain, fevers, and elevated inflammatory markers. An abdominal computed tomography scan showed appendicitis and an electrocardiogram showed third-degree heart block. He underwent successful implantation of a dual-chamber permanent pacemaker and was scheduled for elective appendectomy. This case illustrates a state of system-wide inflammation that has been described mainly in pediatric patients with SARS-CoV-2 known as multisystem inflammatory syndrome in children (MIS-C). Recognition of this syndrome is crucial as it has potential diagnostic and therapeutic implications that can improve outcomes.

## Introduction

The manifestations of COVID-19, caused by SARS-CoV-2, are wide and varied. Recently, there has been a rise in pediatric SARS-CoV-2 cases with systemic involvement [1]. The recognition of the extrapulmonary manifestations of this disease is of utmost importance for diagnosis and management. We present a case of a young man who presented with SARS-CoV-2 symptoms, followed by abdominal pain. He was diagnosed with concurrent radiologically proven appendicitis and complete heart block.

## Case presentation

A 19-year-old man with a past medical history notable for Wolff-Parkinson-White (WPW) syndrome status post unsuccessful radiofrequency ablation (RFA) of the right anterior/anteroseptal accessory pathway complicated by possible injury to atrioventricular (AV) node and Kawasaki disease in childhood presented with SARS-CoV-2 infection, appendicitis, and complete heart block. He was in his usual state of health when he developed subjective fevers, chills, productive cough, and loose bowel movements. He underwent SARS-CoV-2 reverse transcription polymerase chain testing (RT-PCR) testing two days later and was notified of a positive test result. Three days post symptom onset, the patient started having left lower quadrant abdominal pain radiating to the right lower quadrant. This was associated with nausea, vomiting, and dysuria. He presented to the Emergency Department (ED) due to pain severity. He denied any chest pain or shortness of breath and endorsed that he was not vaccinated against SARS-CoV-2. 

**Figure 1:**
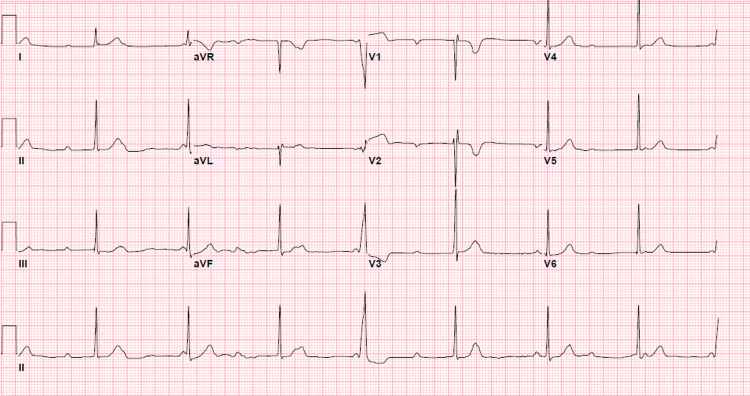
EKG showing complete heart block with a junctional escape ventricular rhythm at a rate of 46 beats per minute and a dissociated atrial rate of 78 beats per minute.

**Figure 2:**
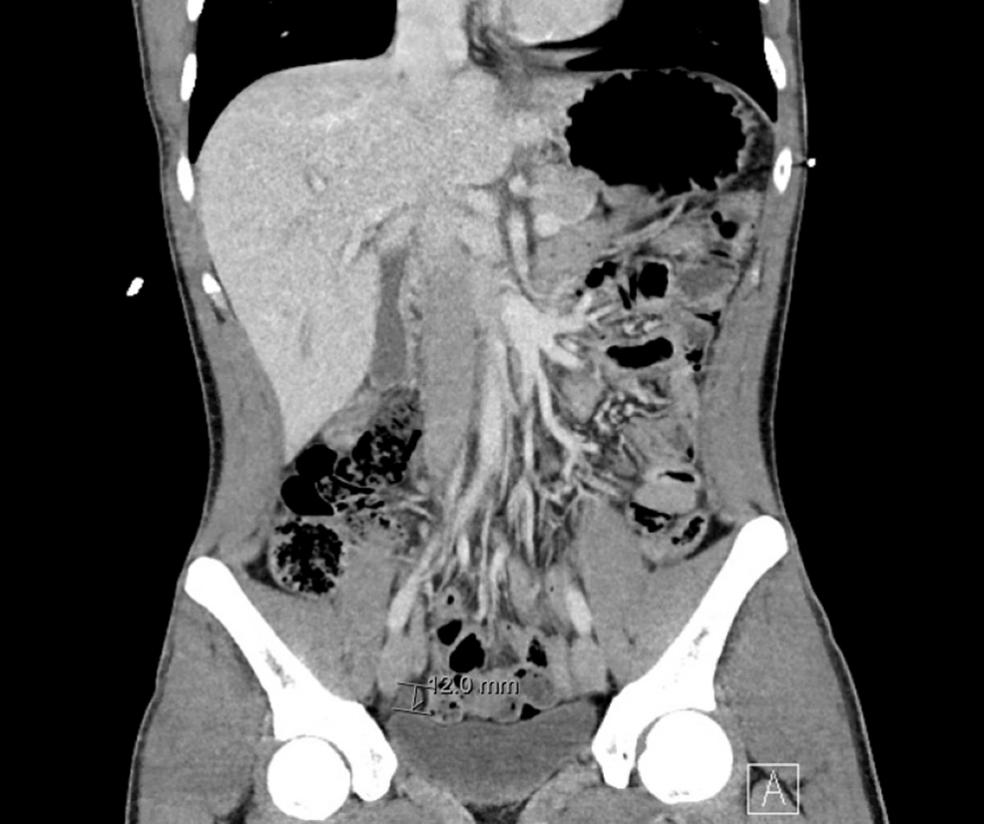
CT scan of the abdomen showing dilated appendix with fluid-filled lumen along with intraluminal appendicolith suggestive of appendicitis.

In the ED, his temperature was 37.1 C, his heart rate was 46 bpm, his blood pressure was 129/75, and his oxygen saturation was 99% on room air. Physical exam was remarkable for bradycardia with normal S1 and S2 heart sounds, clear air entry bilaterally in the lung fields, and tenderness to palpation in the right lower quadrant of the abdomen with rebound tenderness, and a hyperpigmented rash on the lips without mucosal involvement. An electrocardiogram was performed showing a complete heart block with a junctional escape rhythm at a ventricular rate of 46 BPM and a dissociated atrial rate of 78 BPM as shown in Figure 1. Labs were unremarkable without evidence of leukocytosis, kidney or liver injury. Troponin, lipase, thyroid-stimulating hormone, and lactate were within normal limits. C-reactive protein was elevated at 3.21 mg/dL. A computerized tomography (CT) scan of the abdomen and pelvis showed signs of acute appendicitis without periappendiceal abscess or free air as shown in Figure 2.

Further review of the patient’s history revealed that he had been diagnosed with WPW two years prior to presentation. He underwent RFA of the right anterior/anteroseptal accessory pathway outside of the United States and was told the procedure was unsuccessful. He reportedly underwent electrophysiology mapping and hopeful repeat RFA a year later; however, he was told that there may have been an injury to the AV node from the prior procedure. A year later, he presented to the cardiology clinic for noted bradycardia on his smartwatch to HR of 40s and chest discomfort at night. A cardiac event monitor revealed occasional episodes of paroxysmal atrial fibrillation and no episodes of AV block. A permanent pacemaker was not recommended at that time. He was not taking any medications regularly including AV nodal blocking agents.

**Figure 3:**
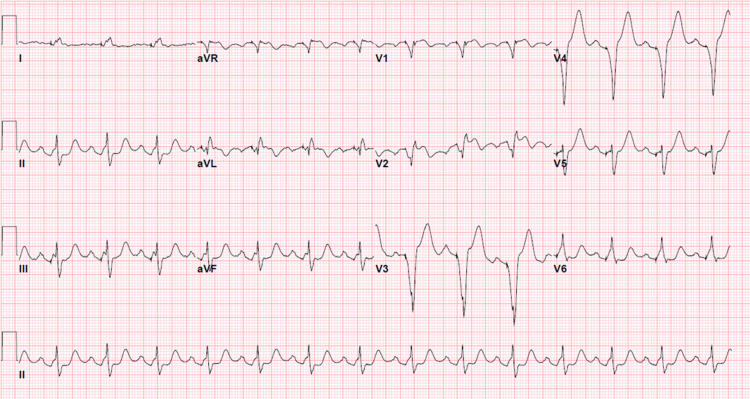
EKG showing atrial-sensed, ventricular paced rhythm after permanent pacemaker (PPM) implantation at a rate of 85 beats per minute.

Given bradycardia in the ER, he received atropine with an improvement of the heart rate to 105 bpm. He was also started on IV piperacillin-tazobactam. The patient was admitted to the Intensive Care Unit for further monitoring. Appendicitis was managed conservatively with antibiotics. On telemetry monitoring, he was found to be in intermittent complete heart block and Mobitz type 2 conduction. His initial response to atropine suggested intermittent conduction through the accessory pathway. He remained hemodynamically stable and did not require transcutaneous or transvenous pacing. From a SARS-CoV-2 perspective, he was only symptomatic with mild upper respiratory infection (URI) symptoms and no oxygen requirement. A dual-chamber permanent pacemaker (PPM) was successfully implanted after the patient recovered from SARS-CoV-2 and he was scheduled for follow-up for elective appendectomy. His EKG following PPM implantation is shown in Figure 3.

## Discussion

We present a case of symptomatic SARS-CoV-2 infection coinciding with appendicitis and complete heart block in a young patient. The patient initially presented with viral URI symptoms and a positive SARS-CoV-2 test followed by abdominal pain. This led to a radiologically confirmed diagnosis of appendicitis as well as evidence of severe inflammation. Moreover, the admission EKG showed third-degree AV block as the underlying rhythm for the presenting bradycardia.

The association of SARS-CoV-2 with cardiac manifestations has been described since the early days of the pandemic. The most common reported associations are acute coronary syndromes, myocarditis, and ventricular arrhythmias. Brady-arrhythmias are much less reported as associations most likely due to under-reporting [2].

In a case series by Chinitz et al., seven patients with no pertinent cardiac history presented with clinically significant bradyarrhythmias in the setting of SARS-CoV-2 infection. In all patients, there was multi-organ involvement and elevation of inflammatory markers. Two of the seven patients had complete heart block similar to the patient presented in this case. Five of the seven patients died of complications of SARS-CoV-2 in the hospital or within three months of discharge suggesting a high mortality rate associated with the concurrence of both diagnoses [3].

In another report, a 10-year-old child presented with SARS-CoV-2, elevation of inflammatory markers, and complete heart block [4]. There are multiple postulations for the possible mechanisms through which SARS-CoV-2 can induce AV nodal dysfunction including inflammation and edema of myocardial tissue through direct viral or immunogenic mechanisms.

There are multiple case reports describing the concurrent diagnosis of SARS-CoV-2 infection with appendicitis. This is a result of retrospective analysis of patients admitted with appendicitis and review of SARS-CoV-2 status. For example, a study by Prichard et al. showed an increase in the number of appendicitis cases compared to the number of cases over the past three years. This is in conjunction with the likelihood of having clinically asymptomatic SARS-CoV-2 that is found on periprocedural testing which might suggest an association between SARS-CoV-2 and appendicitis [5]. Moreover, the pathophysiology of viral infections potentially causing appendicitis has been hypothesized to be related to lymphoid hyperplasia causing an appendicular obstruction which can be the underlying pathophysiology of SARS-CoV-2 infection possibly leading to appendicitis [6].

Organ manifestations of SARS-CoV-2 have previously been described in the literature. Multisystem inflammatory syndrome in children (MIS-C) is one such example. The CDC defines MIS-C as occurring in an individual aged <21 years of age with the presentation including fever, laboratory evidence of inflammation and clinically severe illness including multisystem organ (more than two) involvement, and a positive SARS-CoV-2 test within four weeks prior to presentation [7]. This syndrome applies to the case described in this article. The interesting scenario, in this case, is that the patient had already had a pre-existing history of AV nodal conduction disease. It is unclear whether this was the substrate for developing complete heart block especially because event monitoring prior to presentation did not detect any AV nodal dysfunction. It is more likely that this was a result of SARS-CoV-2 infection as opposed to natural disease course after the unsuccessful ablation for Wolff-Parkinson-White. The clinical implication, in this case, is that management of MIS-C has recently involved the use of intravenous immunoglobulins (IVIG), steroids, and biologic agents such as anakinra (an IL-1ß inhibitor) [8]. While our patient did not exhibit severe inflammatory symptoms requiring the use of IVIG/biologics and steroids were not feasible due to appendicitis, it is important to recognize the available treatment options for patients presenting with such symptoms.

## Conclusions

SARS-CoV-2 remains a challenging illness with multiple complications that are still being researched. The cardiac and gastrointestinal manifestations of SARS-CoV-2 are important to recognize and treat appropriately. The constellation of multiple system involvement can pose a diagnostic challenge to clinicians; therefore, increased reporting of syndromic manifestations is required.

## References

[1] (2022). Multisystem Inflammatory Syndrome in Children (MIS-C) Associated with Coronavirus Disease 2019 (COVID-19). https://emergency.cdc.gov/coca/calls/2020/callinfo_051920.asp.

[2] Babapoor-Farrokhran S, Batnyam U, Wiener PC, Kanjanahattakij N, Khraisha O, Amanullah A, Mainigi SK (2020). Atrioventricular and sinus node dysfunction in stable COVID-19 patients. SN Compr Clin Med.

[3] Chinitz JS, Goyal R, Harding M (2020). Bradyarrhythmias in patients with COVID-19: marker of poor prognosis?. Pacing Clin Electrophysiol.

[4] Assaad IE, Hood-Pishchany MI, Kheir J (2022). Complete heart block, severe ventricular dysfunction and myocardial inflammation in a child with covid-19 infection. JACC: Case Rep.

[5] Prichard C, Canning M, McWilliam-Ross K, Birbari J, Parker W, Wasson L, Hollingsworth JW (2021). Case series of acute appendicitis association with SARS-CoV-2 infection. BMC Infect Dis.

[6] Alder AC, Fomby TB, Woodward WA, Haley RW, Sarosi G, Livingston EH (2010). Association of viral infection and appendicitis. Arch Surg.

[7] Feldstein LR, Rose EB, Horwitz SM (2020). Multisystem inflammatory syndrome in U.S. children and adolescents. N Engl J Med.

[8] McArdle AJ, Vito O, Patel H (2021). Treatment of multisystem inflammatory syndrome in children. N Engl J Med.

